# Restricting Synaptotagmin‐3 Internalization Mitigates Cerebral Ischemia/Reperfusion Injury by Curtailed Neuronal Apoptosis and Microglial Re‐Programming

**DOI:** 10.1002/cns.70815

**Published:** 2026-03-05

**Authors:** Hua Xu, Hongbo Mi, Rong Cheng, Tiantian Gui, Fangzhou Hu, Yi Yang, Jian Cheng, Qun Xue

**Affiliations:** ^1^ Department of Neurology First Affiliated Hospital of Soochow University Suzhou China; ^2^ Department of Neurology Jintan Affiliated Hospital of Jiangsu University, Changzhou Jintan First People's Hospital Changzhou China; ^3^ Jiangsu Key Laboratory of Neuropsychiatric Diseases & College of Pharmaceutical Sciences, Soochow University Suzhou China; ^4^ Department of Pathology Jintan Affiliated Hospital of Jiangsu University, Changzhou Jintan First People's Hospital Changzhou China

**Keywords:** apoptosis, cerebral ischemia/reperfusion injury, microglial reprogramming, neuroinflammation, neuroprotection, Synaptotagmin‐3

## Abstract

**Aims:**

Ischemic stroke is a major global cause of disability and death. Synaptotagmin‐3 (Syt3), a synaptic calcium sensor, exacerbates ischemic injury by promoting pathological glutamate release. This study investigated the potential neuroprotective effect of restriction of Syt3 internalization on neuronal apoptosis and microglial reprogramming following ischemia/reperfusion (I/R).

**Methods:**

The effects of Tat‐GluA2‐3Y (3Y), a cell‐permeable peptide inhibitor of Syt3 internalization, were studied in a mouse model of middle cerebral artery occlusion/reperfusion (MCAO/R) and in oxygen–glucose deprivation/reperfusion (OGD/R)‐induced HT22 and BV2 cells.

**Results:**

The peptide 3Y treatment significantly reduced infarct volume, brain edema, and improved neurological deficits. Additionally, it reduced neuronal apoptosis and also inhibited neuroinflammation by down‐regulating the level of IL‐1b, TNF‐a, and iNOS but up‐regulating the level of TGF‐b1 and Arg‐1. Single‐nucleus RNA sequencing of the peri‐infarct region revealed a dual mechanism: 3Y suppressed pro‐apoptotic gene programs in neurons and facilitated the transformation between different phenotypes of microglia via the cAMP pathway from a pro‐inflammatory to an anti‐inflammatory and immune‐protective state. In vitro, 3Y attenuated oxygen–glucose deprivation‐induced neuronal death and instructed microglia to adopt a protective, TGF‐β1‐secreting phenotype.

**Conclusions:**

Collectively, our findings establish the inhibition of Syt3 internalization as a novel therapeutic strategy that concurrently protects neurons and reprograms the microglial immune response, offering a promising dual‐mechanism approach for acute stroke therapy.

## Graphical abstract

Restriction of Syt3 internalization by peptide 3Y conferred robust neuroprotection against cerebral ischemia/reperfusion injury. Mechanistically, 3Y suppressed neuronal apoptosis fundamentally, and additionally, these salvaged neurons orchestrated and remodulated the transformation of surrounding microglia toward an anti‐inflammatory and immunoregulatory‐oriented direction to mitigate neuronal damage immediately.

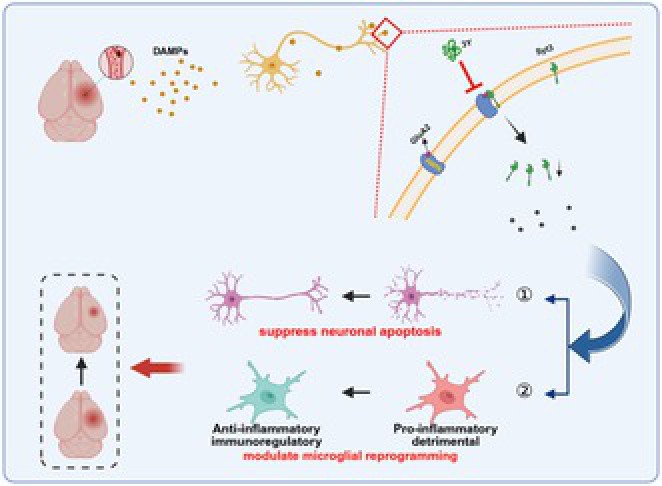

## 1 Introduction

Ischemic stroke is a devastating cerebrovascular disease and a leading threat to human health worldwide, characterized by high rates of morbidity, disability, and mortality [1]. For over a decade, the principal therapeutic strategies for restoring blood flow to the ischemic area have been thrombolysis with recombinant tissue‐type plasminogen activator (rt‐PA) and, more recently, endovascular thrombectomy [2]. However, successful recanalization does not always lead to clinical improvement. In many cases, patients experience aggravated symptoms, such as malignant brain edema and fatal hemorrhagic transformation, following reperfusion. These secondary, delayed injuries are collectively known as cerebral ischemia–reperfusion (I/R) injury [3]. Despite the development of numerous neuroprotective compounds over the past few decades [4], unfortunately, few have proven effective in clinical settings. This failure suggests that strategies focusing exclusively on neuronal targets are insufficient for achieving optimal post‐injury outcomes [5]. Therefore, greater efforts are needed to discover new molecular targets and deepen our exploration of the underlying mechanisms.

Synaptotagmin‐3 (Syt3) is one of the 17 mammalian synaptotagmin isoforms, which are evolutionarily conserved integral membrane proteins involved in trafficking. They are characterized by an N‐terminal transmembrane region (TMR), a variable linker, and 2 C‐terminal C2 domains, and play key roles in processes such as neurotransmitter release [6, 7, 8]. Syt3 is the third most abundant isoform in the brain, with prominent expression in the cortex, cerebellum, brain stem, and hippocampus [7]. It exhibits high calcium affinity due to the strong Ca^2+^‐binding capacity of its C2 domains [8]. Under physiological conditions, Syt3 regulates synaptic strength. Postsynaptic Syt3 facilitates the calcium‐dependent internalization of GluA2‐containing AMPA receptors, dampening synaptic transmission [6], while presynaptic Syt3 accelerates vesicle replenishment to promote short‐term facilitation [9]. Given its calcium sensitivity, Syt3 is also poised to play critical roles under pathological conditions like cerebral I/R, where intracellular Ca^2+^ levels rise dramatically. This Ca^2+^ influx likely triggers Syt3‐mediated AMPAR endocytosis, contributing to neuronal dysfunction. Supporting this, the peptide Tat‐GluA2‐3Y (3Y), which disrupts the Syt3‐GluA2 interaction, was shown to protect neurons from oxygen–glucose deprivation and reoxygenation (OGD/R) in vitro [10]. Our laboratory further demonstrated that 3Y inhibits GluA2‐containing AMPAR endocytosis and confers neuroprotection in vivo [11]. These findings establish 3Y as a potent tool for probing Syt3 function.

However, a critical question remains: does the neuroprotection afforded by inhibiting Syt3 internalization with 3Y also involve modulating microglial activation phenotypes after cerebral I/R injury? The role of microglia, the innate immune sentinels of the CNS, is crucial in this context. Following I/R injury, microglia rapidly accumulate at the lesion site and undergo significant transformations [12]. While their morphological progression follows a sequence [13], their functional states are highly diverse, moving beyond the simplistic M1/M2 dichotomy [14]. Single‐cell RNA sequencing has revealed a remarkable spectrum of microglial subpopulations with unique transcriptional signatures after stroke [15, 16]. These cells often co‐express both pro‐inflammatory and anti‐inflammatory modules [17], making the therapeutic strategy of shifting their balance toward a protective, anti‐inflammatory state highly promising for promoting recovery [18].

Therefore, in this study, we hypothesize that inhibition of Syt3 internalization by 3Y confers neuroprotection not only directly but also by modulating microglial phenotype shifts. We employed a mouse model of middle cerebral artery occlusion/reperfusion (MCAO/R) and an in vitro OGD/R model to investigate the effects of 3Y on neuronal survival and microglial polarization. Furthermore, we performed single‐cell RNA sequencing on peri‐infarct tissue to comprehensively characterize the microglial response and provide a mechanistic rationale for our hypothesis.

## 2 Material and Methods

### 2.1 Animals Housing, MCAO/R Model and Drug Administration

Adult male C57BL/6 mice (8–10 weeks, weight 24 ± 2 g) were purchased from Zhejiang Vital River Laboratory Animal Technology Co. Ltd. (Zhejiang, China) and were housed 8 per cage for 1 week to adapt to the new environment before experiment. All mice were kept on a 12 h light/dark cycle with free access to water and food in 23°C ± 1°C room temperature (humidity 50% ± 5%) except for a period of fasting 12 h before the operation. Efforts were made to minimize the usage and suffering of mice, and all the mice were randomized for the research and procedures. The operator was blinded to the experimental design and data analysis.

Focal cerebral ischemia model was established using MCAO method as previously described [19]. In brief, overnight‐fasted mice were anesthetized with isoflurane and fixed in a supine position on a heating pad to maintain the body temperature in the range of 37.0°C ± 0.5°C. The right common carotid artery (CCA), external carotid artery (ECA), and internal carotid artery (ICA) were exposed and isolated discretely. Subsequently, a silicone nylon filament with a rounded tip (0.21 mm in diameter) was inserted into the ICA through the distal ligated ECA to block the blood flow in the middle cerebral artery (MCA). Cerebral blood flow (CBF) was monitored by laser Doppler flowmetry (PeriFlux System 5000; Perimed Sweden), and successful MCAO was defined as the CBF declining 75% from baseline. After 60 min of occlusion, the nylon filament was gently withdrawn for reperfusion. Mice were then placed in heated cages to recover from anesthesia. After fully awake, the neurological behavior of all the MCAO/R mice was examined using a Longa score method, and mice with a score of 1 to 3 were included in the study [20]. Sham mice underwent the identical procedure without filament insertion.

Mice with a successful ischemia–reperfusion model were stochastically assigned into two groups. One group received peptide 3Y at 1 h after reperfusion and intraperitoneally once daily. The dosage of peptide 3Y (Tat‐GluA2‐3Y, GL Biochem Ltd., China) dissolved in saline was 5 mg/Kg as previously described [11]. Meanwhile, another group injected the same volume of saline (vehicle).

### 2.2 Cell Culture, OGD/R Model and Conditioned Medium Administration

Mouse microglial cells (BV2) donated by Professor Jian Cheng's lab (Jiangsu Key Laboratory of Neuropsychiatric Diseases, Suzhou, China) and mouse hippocampal neurons (HT22) obtained from PhD. Jiansheng Liu (Donghua University, Shanghai, China) were cultured in Dulbecco's modified Eagle's medium (DMEM, Corning, USA) supplemented with 10% fetal bovine serum (Sigma‐Aldrich, MO, USA) and 1% penicillin–streptomycin (Hyclone, Logan, UT, USA). Both of them were incubated at 37°C in a humidified 5% carbon dioxide atmosphere, with the medium changed daily or every 2 days.

In order to mimic I/R injury in vitro, cells were exposed to OGD/R as previously reported [21]. Briefly, the medium was replaced with D‐glucose‐free DMEM (Corning) and incubated at 37°C in a hypoxic incubator (94% nitrogen and 5% carbon dioxide) for 2 h to simulate OGD, and then the cells were transferred into a normal incubator with high glucose‐containing DMEM medium for an additional 22 h to mimic reoxygenation.

After 1 h of reoxygenation, 3Y (2 mM) [22] or/and H89 (10 mM) [23] was added in the culture medium of HT22 cells and the conditioned medium (CM) was collected 21 h later for further experiments. To investigate the effect of damaged neuronal microenvironment on post‐I/R microglial polarization, we performed further experiments according to previously published methods [24]. BV2 cells were subjected to OGD injury for 2 h, and then incubated with CM collected from OGD/R stimulated HT22 cells with different treatments at the start of 22 h reoxygenation.

### 2.3 Tissue and Cell Preparation

Three days after MCAO/R, anesthetized mice were sacrificed by transcardiac perfusion with pre‐cooled phosphate‐buffered saline (PBS) after peripheral blood was obtained. All blood samples obtained were centrifuged within 30 min at 1000 g for 15 min at room temperature (RT) to collect the supernatant, which was further centrifuged at 13,000 g for 3 min to collect cell‐free plasma and stored at −80°C for ELISA. The brains for immunostaining were dissected and fixed with 4% paraformaldehyde (PFA, 158127 MSDS, Sigma) overnight at 4°C, and then after gradient dehydration, brain tissues were embedded in paraffin and sectioned with 5 mm thickness. Peri‐infarct tissues for single‐cell sequencing were harvested directly by decapitation 24 h after reperfusion. The brain tissues from MCAO/R mice and sham ones were dissected and frozen at −80°C for quantitative real‐time polymerase chain reaction (qRT‐PCR), western blotting, and ELISA. BV2 cells cultured on coverslips were fixed with 4% PFA for 20 min at RT and stained directly. Brain samples and BV2 cells were homogenized in the lysis buffer on a rotary shaker for 90 min on ice, centrifuged at 1500 rpm for 15 min at 4°C to collect the supernatant for ELISA. Proteins for western blotting were extracted from brain tissues using a commercial protein extraction kit (Beyotime Biotech, Wuhan, China) and the concentration was determined using a Nanodrop Spectrophotometer (ND‐2000; ThermoFisher, Carlsbad, CA) at an optical density (OD) of 280 nm. Total RNA for qRT‐PCR was extracted from peri‐infarct brain or BV2 cells using the TRIzol method and the spectrophotometric analysis at OD 260/280 nm (> 1.8) was used to ensure the purity and quantity of RNA. Last, the supernatant of HT22 cell cultures, as conditioned culture medium, was harvested and centrifuged at 12,000 rpm for 10 min to remove debris sterilely.

### 2.4 Single Nucleus Isolation

To prepare single nucleus suspensions, the peri‐infarct area of ipsilateral hemispheres of MCAO/R mice was rapidly isolated and kept in MACS Tissue Storage Solution (Miltenyi Biotech, 130‐100‐008, Germany). Brain tissues were rapidly transferred to CapitalBio Technology (Beijing, China) for subsequent sample processing and data acquisition. Single nucleic suspension was prepared following the instruction of Nucleic preparation kit (CapitalBio, XS0100101). Briefly, fresh tissue was homogenized in a Dounce homogenizer (MACS RTisochip‐A) in Lysis Buffer with 1 mM DTT and 1 U/μL RNase inhibitor (Thermo Fisher Scientific), and incubated on ice for 5 min. The suspension was collected and filtered through a 40 mm strainer (Miltenyi Biotech, Germany) to remove debris and large clumps. The single nucleus suspension was centrifuged at 500 g for 5 min at 4°C and then sequentially resuspended in 300 mL Lysis buffer and 300 mL RB buffer. Subsequently, the mixture was centrifuged by density gradient to separate the nuclei from cell debris, and the intermediate layer washed with RB buffer was collected. Finally, the cell number and viability were assessed by an automatic cell counter (CauntStar Rigel S2).

### 2.5 Single‐Nucleus RNA‐Seq Library Preparation and Sequencing

The nuclei were loaded into the microfluidic chip of the Chip A Single Cell Kit v2.0 (MobiDrop, S050100201) to generate droplets with MobiNova‐100 (MobiDrop, A1A40001). Each nucleus was involved in a droplet that contained a gel bead linked with up to millions of oligos (cell unique barcode). After encapsulation, droplets suffered light cut by MobiNovaSP‐100 (MobiDrop, A2A40001) while oligos diffused into the reaction mix. The mRNAs were captured by gel beads containing oligo (dT) in droplets. Following reverse transcription, cDNAs with barcodes were amplified, and a library was constructed using the High Throughput Single Cell 3′ RNA‐Seq Kit v2.0 (MobiDrop (Zhejiang) Co. Ltd., S050200201) and the 3′ Single Index Kit (MobiDrop (Zhejiang) Co. Ltd., S050300201).

After library construction, sequencing was performed on the platform of Illumina NovaSeq 6000 System (CapitalBio Technology, Beijing). Cell Ranger 10X Genomics software was used to perform sample demultiplexing, barcode processing, and generating gene count data for each cell. The cDNA insert was aligned to the GRCm39 reference genome. The feature‐barcode matrices were generated for each sample by counting the valid barcodes and unique molecular identifiers (UMIs). Further analyzes, including quality control, identification of highly variable features, and unsupervised clustering, were performed using Seurat (4.0.4, http://satijalab.org/seurat/) R toolkit.

### 2.6 snRNA‐Seq Data Analysis

For each snRNA‐seq data, high‐quality cells were reserved if the mitochondrial gene expression was less than 10% and the detected genes were between 200 and 5000. The “RunHarmony” function [25] was performed to minimize the technical and biological batch effects among individuals and experiments. The top 4000 genes were used to perform principal component analysis (PCA), and the first 30 PCs were utilized to reduce the high‐dimensional data to two‐dimensionality. Based on the expression distribution of conventional biomarkers for each cell type, the cells were annotated into four major cell types, including neurons, microglia, oligodendrocytes, and astrocytes. The apoptosis‐related genes were obtained from the REACTOME database, and the “AddModuleScore” function was used to calculate the apoptosis scores of each neuron cell. To assess the functional states of microglia in the snRNA‐seq data, we generated gene signatures representative of pro‐inflammatory and anti‐inflammatory responses (commonly referred to as M1‐like and M2‐like states in prior literature). In addition, by utilizing the “AddModuleScore” function in the Seurat package, we assessed the pro‐inflammatory score and anti‐inflammatory score for microglia based on the conventional signatures: pro‐inflammatory: Tnf, Cxcl10, Cd86, Il1a, Il1b, Il6, Ccl5, Irf5, Irf1, Cd40, Ido1, Kynu, Ccr7; anti‐inflammatory: Cd163, Cd209e, Ctsc, Ctsb. According to the application of CellPhoneDB software [26, 27] in investigating the potential functional mechanisms of crucial cell subpopulations [28], we constructed the molecular interaction networks to study the role of neuron cells in mediating the cellular status of other cell populations. The ligand‐receptor pairs with a *p* value < 0.05 were retained for the assessment of the relationship among different cell clusters.

### 2.7 Neurobehavioral Tests

To obtain reliable and reproducible results, the other two experimenters who were trained all the behavioral test protocols but blinded to the experiment design were needed. All mice included in the experiments were subjected to the following behavioral tests. All testing facilities were thoroughly cleaned between each section.

#### 2.7.1 Modified Neurological Severity Score (mNSS)

The paradigm of mNSS was performed as previously described to evaluate the neurobehavioral defect of cerebral ischemic mice [29]. The motor, sensory, reflex, and balance coordination was fully reflected in this test. The score, graded from 0 to 18 (1–6 is classified as mild damage, 7–12 as moderate damage, and 13–18 as severe defects), with higher scores indicating remarkable severity of neurological impairment.

#### 2.7.2 Rotarod Fatigue Test

The rotarod fatigue test was applied to assess the motor coordination in mice, as previously reported [30]. Briefly, mice were placed on an accelerating rotarod, which contains six runways evenly separated by black panels. The instrument system was set to accelerate from 4 to 40 rpm within 5 min. When the mouse fell off, or gripped and stuck around for two successive cycles without attempting to walk on the rod, the trial was stopped and the time would be recorded automatically. All mice were trained three to five times daily for three consecutive days before MCAO/R, each trial lasting for 5 min. Finally, the average time of three tests was measured for each mouse in formal experiments.

#### 2.7.3 Adhesive Removal Test

To evaluate both somatosensory and motor deficits, the adhesive removal test was conducted complying with the protocol of Valentine et al. [31]. A transparent Perspex box (15 cm × 25 cm) and small adhesive tape strips (0.3 cm × 0.4 cm) were utilized in this test. Per mouse was placed in the testing box for a habituation period of 1 min, and next gently removed the mouse from the box and applied the adhesive tape strips with equal pressure on the left forepaw so that they would cover the hairless part of the paw (i.e., the three pads, thenar and hypothenar), and immediately replaced the mouse back with a maximum duration for 2 min to remove the tape. The contact time was defined as the point that the mouse reacted to the presence of the adhesive tape strip, either shaking leftpaw or directly bring the paw to its mouth was adopted. Subsequently, when the mouse removed the adhesive tape from its original position with mouth or the right forelimb, it represented the end of the removal time. The time required to contact and remove both stimuli from each forelimb was recorded. All mice were recommended to train one trial per day for 5 days before surgery.

#### 2.7.4 Foot‐Fault Test

For locomotor assessment, the placement dysfunction of forelimbs presented by mice was examined via modified foot‐fault test [32]. In short, per mouse was placed on an elevated grid apparatus (30 cm length × 30 cm width × 30 cm height) with a grid opening of 2.5 cm^2^, and the behavior of which was videoed bottom up separately. Each test consisted of three trials for 1 min, with an interval of 5 min at least. Foot faults were interpreted when the mouse misplaced its impaired paw (left side), causing the paw to fall through the grid (also called stepping empty). Finally, the total number of steps taken on the grid and the number of errors made on bilateral limbs were recorded by another blinded investigator. The left side foot‐faults (%) = (left error steps − right error steps)/total steps × 100%. All experiment mice were subjected to train 3 times a day for 3 days before surgery.

### 2.8 Cerebral Infarction and Brain Edema

Cerebral infarction was detected by 2, 3, 5‐triphenyl tetrazolium chloride (TTC, Sigma‐Aldrich, St. Louis, Mo, USA) staining. After cervical dislocation, the brains were removed rapidly and sectioned into serial coronal slices (2.0 mm thick) using mouse matrix. Afterward, these sections were immersed in a 1% TTC solution and stained in the dark at 37°C for 10 min, during which the brain slices were flipped over repeatedly to ensure consistent coloring. Subsequently, the sections were fixed in 4% paraformaldehyde (PFA) overnight. The infarcted tissue was stained white and imaged by digital camera. The peri‐infarct area was defined as the region within normal brain tissue 1 mm away from the infarction boundary line. The infarction area was analyzed by Image J software and calculated with the formula: Infarction (%) = (contralateral hemisphere area − ipsilateral nonischemic hemisphere area)/contralateral hemisphere area × 100% [33].

Brain edema was expressed by brain water content applying wet/dry method as previously described [34]. Briefly, the removed brains were immediately divided into ipsilateral hemisphere and contralateral hemisphere, which were placed on paper piece and the wet total weight was measured separately, and the dry weight was measured again after 48 h of heating at 70°C in thermostat box. The weight of paper piece was measured just before the wet weight obtained. The brain water content was calculated as the following formula (wet weight − dry weight)/(wet weight − paper weight) × 100%.

### 2.9 Enzyme‐Linked Immunosorbent Assay (ELISA)

The concentrations of Tumor necrosis factor‐α (TNF‐α), Interleukin‐1b (IL‐1b), Interleukin‐10 (IL‐10), and TGF‐b1 in the ipsilateral brain penumbra of C57BL/6 mice and plasma were determined by corresponding enzyme‐linked immunosorbent assay kits (Absin, Shanghai, China). The procedures were in accordance with the manufacturer's instructions.

### 2.10 Quantitative Real Time Polymerase Chain Reaction (qRT‐PCR)

As previously described [35], qRT‐PCR was performed to measure the levels of cytokines gene. In brief, the total RNA from ipsilateral penumbra and BV2 cells was extracted by the TRIzol Reagent (Thermo Fisher Scientific, USA) and HiScript Q RT SuperMix Kit (Vazyme, China) was used to reverse‐transcribe RNA into cDNA. Subsequently, the reactions were performed in the Bio‐Rad CFX96 Real‐Time system utilizing the UltraSYBR Mixture (Comwin Biotech, China). The mRNA levels of *Tnfa*, *Il1b*, *Il10*, and *Tgfb1* were presented as 2^−ΔΔCT^ and normalized against glyceraldehyde‐3‐phosphate dehydrogenase (*Gapdh*). The primer sequences were synthesized by EnzyArtisan (Shanghai, China) as described in Table S1.

### 2.11 Western Blot

The total proteins in ipsilateral brain penumbra were extracted with RIPA lysis buffer (Beyotime Biotechnology, Shanghai, China), and after centrifuged with 12,000 g at 4°C for 30 min, the supernatant was collected. The membrane proteins were also isolated according to the instructions of the manufacturer. The protein concentrations both in tissue and membrane were detected by BCA protein assay kit (Beyotime Biotechnology, China). Subsequently, the protein samples were separated through 10% sodium dodecyl sulfate‐polyacrylamide gel electrophoresis (SDSPAGE), and eventually transferred onto polyvinylidene difluoride membranes (PVDF; Millipore, USA). The membranes were blocked with 5% non‐fat powder milk in Tris‐buffered saline containing Tween‐20 (TBST) at RT for 1 h and then incubated overnight at 4°C with the following primary antibodies: anti‐Syt3 (1:10000, NBP1‐19320, Novus), anti‐caspase3 (1:2000, 19677‐1‐AP, Proteintech), anti‐Bax (1:1000, 50599‐2‐Ig, Proteintech), anti‐Bcl‐2 (1:1000, 26593‐1‐AP, Proteintech), anti‐iNOS (1:1000, 13120, CST), anti‐Arg‐1 (1:1000, 93,668, CST), anti‐b‐actin (1:2000, Affinity Biosciences), anti‐GAPDH (1:100000, 60004‐1‐Ig, Proteintech), anti‐ Na+ ‐K+ ‐ATPase α1 (ATP1A1, 1:5000, 55187‐1‐AP, Proteintech), anti‐Histone H3 (1:5000, 17168‐1‐AP, Proteintech), phospho‐PKA Thr198 (p‐PKA, 1:1000, AF7246, Affinity), PKA (1:1000, AF7746, Affinity), phospho‐CREB Ser133 (p‐CREB, 1:1000, 9198, CST) and CREB (1:1000, 9197, CST). After washing with TBST or PBST, the PVDF membranes were subsequently incubated with the appropriate secondary antibodies at room temperature for 2 h. Bands were observed with an enhanced chemiluminescence kit (Millipore, USA) using a gel imaging system (Bio‐Rad, USA). The quantification of individual protein bands was performed using densitometry analysis with the ImageJ software.

### 2.12 Fluoro‐Jade C (FJC) Staining

The evaluation of degenerating neurons was applied by FJC staining [36]. The procedures were followed by the FJC kit (Biosensis, South Australia) protocol. Briefly, after dewaxed and hydrated, brain sections were rinsed in 1× KMNO4 (0.06%) for 10 min and next placed in distilled water for 2 min and subsequently immersed in a working cocktail solution containing 1× Fluoro Jade C (0.0001%) and 1× DAPI in the dark for 10 min at room temperature. Following 3 times of 1‐min rinse with deionized water, the sections were dried in an incubator (50°C–60°C) for 8 min. Finally, the dried brain sections were transferred into clean xylene solution for 5 min and sealed with an anhydrous and glycerine‐free styryl sealer DPX.

### 2.13 Immunofluorescence

Fixed BV2 cells (cultured on coverslips) by 4% PFA were permeabilized with 0.2% Triton X‐100 (Sigma‐Aldrich, USA) for 10 min at RT. Then, the deparaffinized and antigen‐repaired brain sections and treated BV2 cells were blocked with 5% bovine serum albumin (Sigma‐Aldrich, USA) for 30 min at 37°C and incubated with rabbit anti‐NeuN (1:500, 24307, CST), mouse anti‐Syt3 (1:100, NBP1‐19320, Novus), mouse anti‐ionized calcium‐binding adapter molecule‐1 (Iba‐1, 1:500, ab283319, Abcam), rabbit anti‐iNOS (1:500, 13120, CST), or rabbit anti‐Arg‐1 (1:200, 93668, CST) antibodies overnight at 4°C. After 3 times rinsing with PBS, the tissues and cells were stained with corresponding secondary antibodies conjugated to either Alexa Fluor 488, Alexa Fluor 594 or Alexa Fluor 546 (1:500, Invitrogen) in the dark at 37°C for 1 h. Nuclei were counterstained with 4′,6‐diamidino‐2‐phenyl‐indole (DAPI, Abcam).

### 2.14 Terminal Deoxynucleotidyl Transferase‐Mediated dUTP Nick End Labeling (TUNEL) Assay

Immunofluorescent double staining of neuronal nuclei (NeuN) combining terminal deoxynucleotidyl transferase‐mediated dUTP nick end labelling (TUNEL) was performed to quantify neuronal apoptosis in penumbra. Briefly, the fixed brain tissues were blocked and incubated with rabbit anti‐NeuN antibody (1:500, 24307, CST) at 4°C overnight, and next stained with Alexa Fluor 488‐conjugated secondary antibody (1:500; Invitrogen). Thereafter, the sections were subjected to TUNEL staining using the Elabscience One‐step TUNEL In Situ Apoptosis Kit (Elabscience Biotechnology Co. Ltd., China). All the following steps were performed in the dark and humid environment. Finally, the sections were coverslipped with the DAPI counterstain.

### 2.15 Cell Counting

Images were captured under a fluorescence microscope (Nikon, Japan). Three coronal brain sections per animal were included, and three fields within the penumbra in each section were randomly selected. The positive cells were assessed at a magnification of 200×. Fluorescently labeled cells were performed manually using Image J software (National Institutes of Health) by another blinded investigator.

### 2.16 Statistical Analysis

The data were statistically analyzed using IBM SPSS 20.0 software, and the graphic representations of quantitative data were performed by GraphPad Prism 9.0 software. Fisher's exact test was used to analyze the bioinformatic data between 3Y‐ and vehicle‐treated groups. The Student's *t*‐test were used to determine the differences between two groups. To evaluate one factor among three groups, one‐way analysis of variance, followed by Tukey's post hoc test was used. For behavioral data, Abnormal was defined when the value was 2 SEM deviated from that of the controls. All data were expressed as mean ± SEM, and differences were considered significant at *p* < 0.05.

## 3 Results

### 3.1 Peptide 3Y Alleviated Neurobehavioral Deficits in Mice Subjected to MCAO/R

**FIGURE 1:**
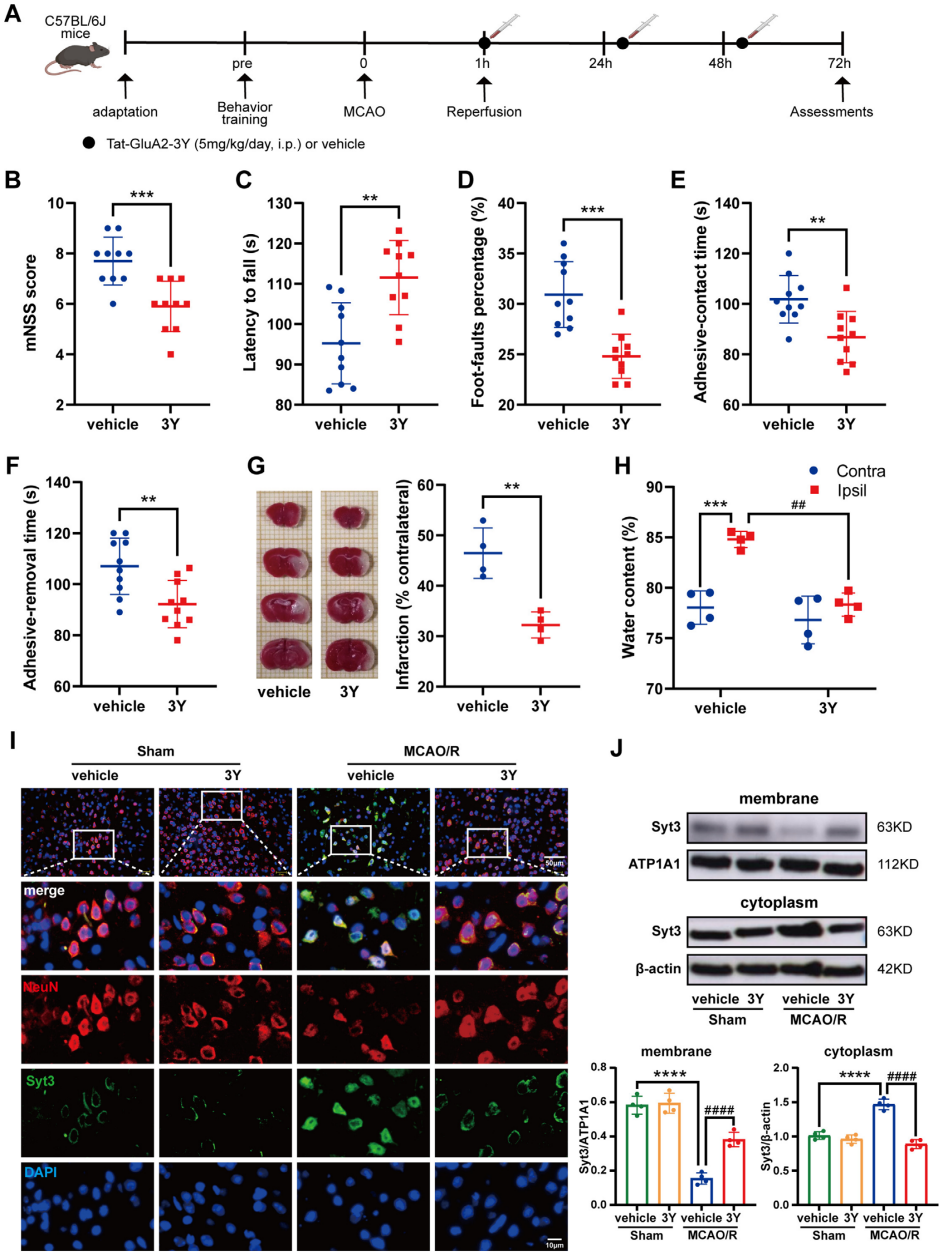
Effect of peptide 3Y on neurobehavior and Syt3 expression in MCAO/R mice. (A) Flow chart of the experiment design. (B–F) Neurological dysfunction was evaluated by mNSS (B), rotarod fatigue test (C), foot fault assays (D) and adhesive removal test (E, F) in ischemic mice. **p < 0.01, ***p < 0.001, *n* = 10. (G) TTC staining 24 h after reperfusion. **p < 0.01, *n* = 4. (H) The statistical chart of water content between ipsilateral hemisphere and contralateral hemisphere 24 h after reperfusion. ***p < 0.001, ipsilateral hemisphere versus contralateral hemisphere; ^##^ *p* < 0.01, the ipsilateral water content in 3Y group versus the one in vehicle group, *n* = 4. (I) Immunofluorescence co‐localization of Syt3 in different groups. (J) Western blot diagram and the statistical charts of Syt3 in cytoplasm and membrane from peri‐infarct region between Sham and MCAO/R mice. ****p < 0.0001 versus Sham + vehicle group; ^####^ *p* < 0.0001 versus MCAO/*R* + vehicle group, *n* = 4. Data were presented as the mean ± SEM.

Peptide 3Y has been proven to mimic the blocker of Syt3 internalization, thus we utilized 3Y to investigate its therapeutic effects on cerebral I/R injury. To explore the role of Syt3 after I/R injury, we evaluated the neuroprotective effect of 3Y in mice subjected to MCAO/R. And several different behavioral paradigms were used to assess the motor, sensory, balance and reflex activity (experiment design was shown in Figure 1A). 3Y significantly improved the neurological function of MCAO/R mice, as displayed with lower mNSS scores (Figure 1B), longer latency to fall in rotarod fatigue test (Figure 1C), lower percentage in foot‐fault test (Figure 1D) and less contact and removal time in adhesive removal test (Figure 1E,F), but had no substantial effect on the behavior of Sham operated mice. Peptide 3Y treatment significantly reduced cerebral infarction (Figure 1G) and brain edema (Figure 1H) in ipsilateral hemisphere, as compared with mice receiving vehicle buffer. To further examine the expression of Syt3 both in cytoplasm and membrane within peri‐infarct area, western blotting and immunofluorescence co‐localization were utilized 3 days after ischemic model of MCAO/R in mice. Immunostaining intuitively revealed that Syt3 is mainly located on neuron membrane in the Sham group, while a large amount of green fluorescence of Syt3 was seen in the cytoplasm in MCAO/*R* + vehicle group. However, the cytoplasmic immunofluorescence intensity was significantly decreased in MCAO/R + 3Y group (Figure 1I). Simultaneously, the data from western blot showed that the expression of cytoplasm Syt3 increased significantly in MCAO/R + vehicle group compared to that in Sham group; instead, the level of membrane Syt3 was lower than Sham + vehicle group, and expectedly, this phenomenon was remarkably reversed by peptide 3Y. Certainly, there was no difference between vehicle‐ and 3Y‐treated groups among Sham operated mice (Figure 1J). These results demonstrated that restriction of Syt3 internalization by peptide 3Y effectively enhanced the recovery of neurological deficits induced by I/R injury.

### 3.2 Peptide 3Y Attenuated Neuronal Apoptosis Within Peri‐Infarct Area In Vivo

**FIGURE 2:**
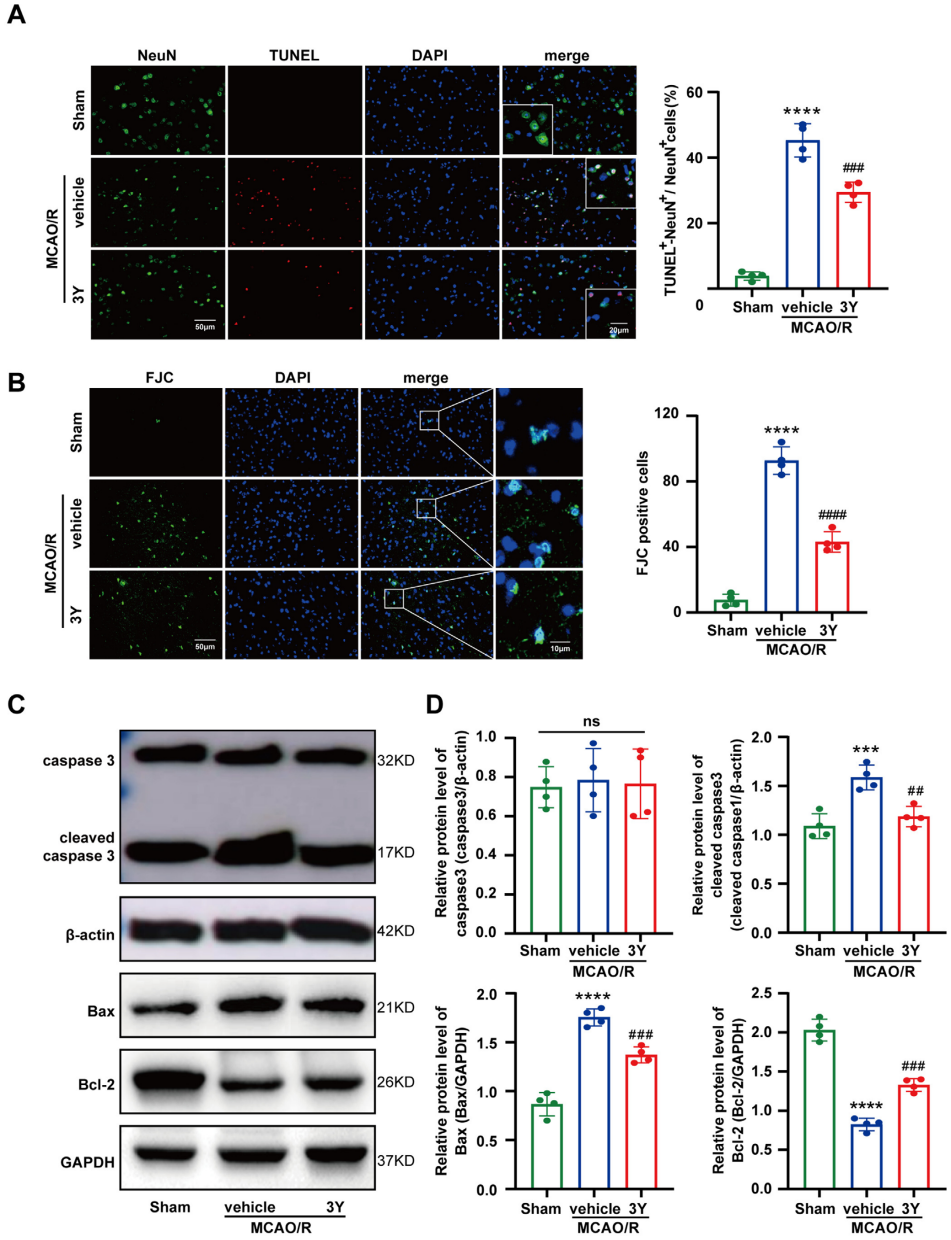
Effects of 3Y on neuronal apoptosis and degeneration after ischemic‐reperfusion injury. (A) Representative images of the colocalization of TUNEL (red) with neurons (NeuN, green) within peri‐infarct area and quantitative analysis of the proportion of TUNEL‐NeuN double positive cells to the total NeuN‐positive cells. Scale bar = 50 mm. (B) The staining and quantitative analysis of FJC (green) 3 days post‐MCAO/R. Scale bar = 50 mm. (C) Representative western blot bands of caspase 3, cleaved caspase 3, Bax and Bcl‐2 in peri‐infarct area. (D) Quantitative analyzes of caspase 3, cleaved caspase 3, Bax and Bcl‐2 protein levels. ***p < 0.001, ****p < 0.0001 vs. Sham group, ^##^ *p* < 0.01, ^###^ *p* < 0.001, ^####^ *p* < 0.0001 vs. MCAO/*R* + vehicle group. Error bars are represented as mean ± SEM. *n* = 4 per group.

Having established that 3Y improves functional recovery, we next sought to determine whether this was associated with enhanced neuronal survival in the ischemic penumbra. As the primary executors of brain function, the preservation of viable neurons is a critical determinant of stroke outcome, and thus we investigated the effect of peptide 3Y on neuron survival. TUNEL and NeuN double staining was performed to evaluate the neuronal apoptosis in the peri‐infarct region following recanalization. Compared with the sham group, the proportion of TUNEL‐NeuN double positive cells to NeuN labeled neurons raised markedly in the vehicle group, while the treatment of peptide 3Y significantly decreased the higher proportion of TUNEL‐NeuN/NeuN ascribed to MCAO/R (Figure 2A). Next, neuronal degeneration was also evaluated by FJC staining. The number of FJC‐positive cells increased significantly in the vehicle group compared with the sham group but was obviously reversed by peptide 3Y (Figure 2B). To confirm the effect of 3Y on cell apoptosis within the peri‐infarct area, western blot analysis showed that peptide 3Y reduced apoptotic proteins of cleaved caspase 3 and Bax but increased the expression of Bcl‐2 compared to the vehicle group. Whereas, 3Y administration had no significant influence on caspase 3 (Figure 2C,D). These results suggested that peptide 3Y exerted protective effects on neuronal apoptosis in MCAO/R mice.

### 3.3 Peptide 3Y Treatment Downregulated Neuronal Degeneration and Apoptosis

**FIGURE 3:**
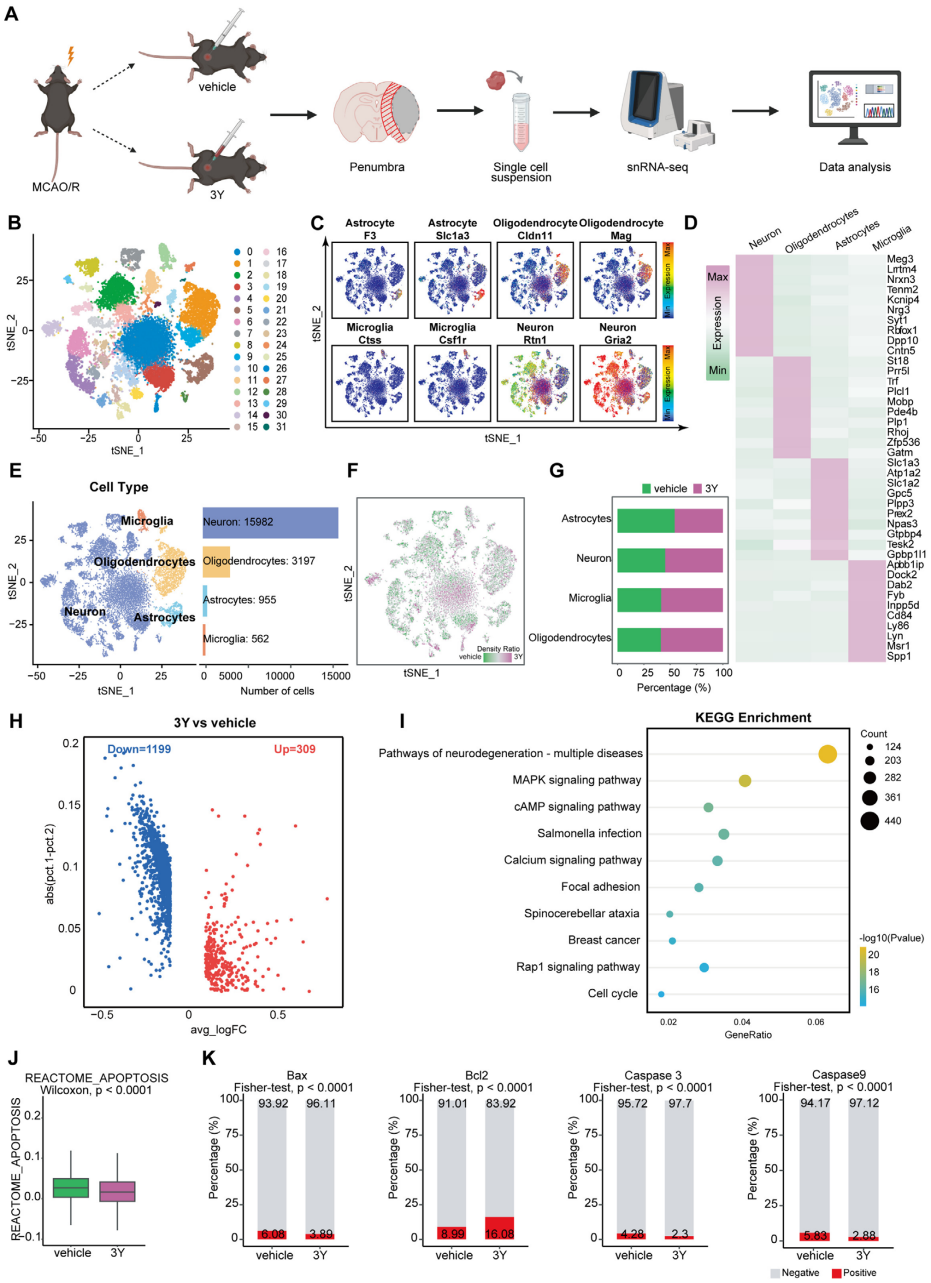
Identification of cell types and gene differential expression analysis of the effects of peptide 3Y on neuron. (A) The flowchart procedure of snRNA‐seq analysis of brain penumbra tissue samples from 3Y‐ and vehicle‐treated mice with MCAO/R. (B) The unsupervised clustering of 20,696 cells. (C) The expression of classic gene markers for each cell subpopulation on the t‐SNE representation. (D) Heatmap for gene expression levels of top 10 cell‐type‐specific genes. (E) Left: T‐SNE visualization of cell types annotated by classical gene markers. Right: Bar plot showing the cell numbers of each cell type. (F) Heatmap showing the distribution density ratio of cells from 3Y‐ and vehicle‐treated mice under MCAO/R. The t‐SNE visualization is split into 200 × 200 bins equally. (G) Proportional histogram depicting the proportion alterations of 4 transcriptionally distinct cell types form 3Y‐ and vehicle‐treated mice. (H) Volcano plot showing the upregulated and downregulated DEGs (log2FC > 0.15, FDR < 0.05) between 3Y‐treated neurons and vehicle neurons. (I) Scatter plot of KEGG pathway enrichment analysis of upregulated DEGs in neurons treated with 3Y. (J) Comparison of apoptosis scores of neurons between 3Y‐ and vehicle‐treated groups from REACTOME database. (K) Stacked histogram showing the expressed percentage of apoptosis‐related genes in neurons from 3Y‐ and vehicle‐treated groups.

Our histological and biochemical data demonstrated that 3Y attenuates neuronal apoptosis. To gain a comprehensive, unbiased understanding of the transcriptional programs underlying this protective effect, we performed droplet‐based snRNA‐seq on peri‐infarct brain tissues from vehicle‐ and 3Y‐treated mice (Figure 3A). After strict quality control, 20,696 cells (vehicle: 9290 cells, 3Y: 11,406 cells) with a mean of 3795–4804 unique molecular identifiers (UMIs) per cell were collected for subsequent analysis. The average number of genes per cell was 1971–1427 among the two groups (Table S2). Next, according to the classic markers of various brain cell types (Figure 3C), the unsupervised clustering and annotated cell types we performed and 32 clusters including neurons (15,982 cells), oligodendrocytes (3197 cells), astrocytes (955 cells), microglia (562 cells) and other types of cells were identified (Figure 3B,E). The specifically expressed top 10 genes of each cell type were identified to further verify the annotation of single‐cell (Figure 3D). It is worth noting that the proportion of microglia and oligodendrocytes in 3Y‐treated mice increased significantly compared with that in the vehicle‐treated group (Figure 3G). Next, the differentially expressed genes (DEGs) were analyzed, among the total 1508 DEGs, 309 were upregulated while 1199 were downregulated in 3Y‐treated neurons compared to vehicle‐treated neurons (Figure 3H). Notably, genes associated with cell growth and anti‐apoptosis such as Mapk1, Akt, and Bcl were upregulated markedly. Subsequently, we applied KEGG pathway enrichment analysis to further verify the upregulated DEGs and found the neurodegeneration‐related pathway, MAPK signaling pathway and cell cycle‐related pathway were highly enriched in neurons delivered with peptide 3Y (Figure 3I). Neurons either to death or survival is crucial to the outcome of the stroke surrounding the infarct area, where cell apoptosis has always been concerned. The results from REACTOME showed the scores of apoptosis in 3Y‐ was lower than those in vehicle‐treated mice (Figure 3J). Specifically, the expression of pro‐apoptotic genes including Bax, Casp3, and Casp9 decreased while the anti‐apoptotic gene Bcl‐2 increased strikingly (Figure 3K). These results indicate that 3Y confers neuroprotection by suppressing neuronal apoptosis following cerebral ischemia–reperfusion.

### 3.4 Peptide 3Y Rebalanced the Microglial Response, Suppressing a Pro‐Inflammatory State and Promoting a Protective State in MCAO/R Mice

**FIGURE 4:**
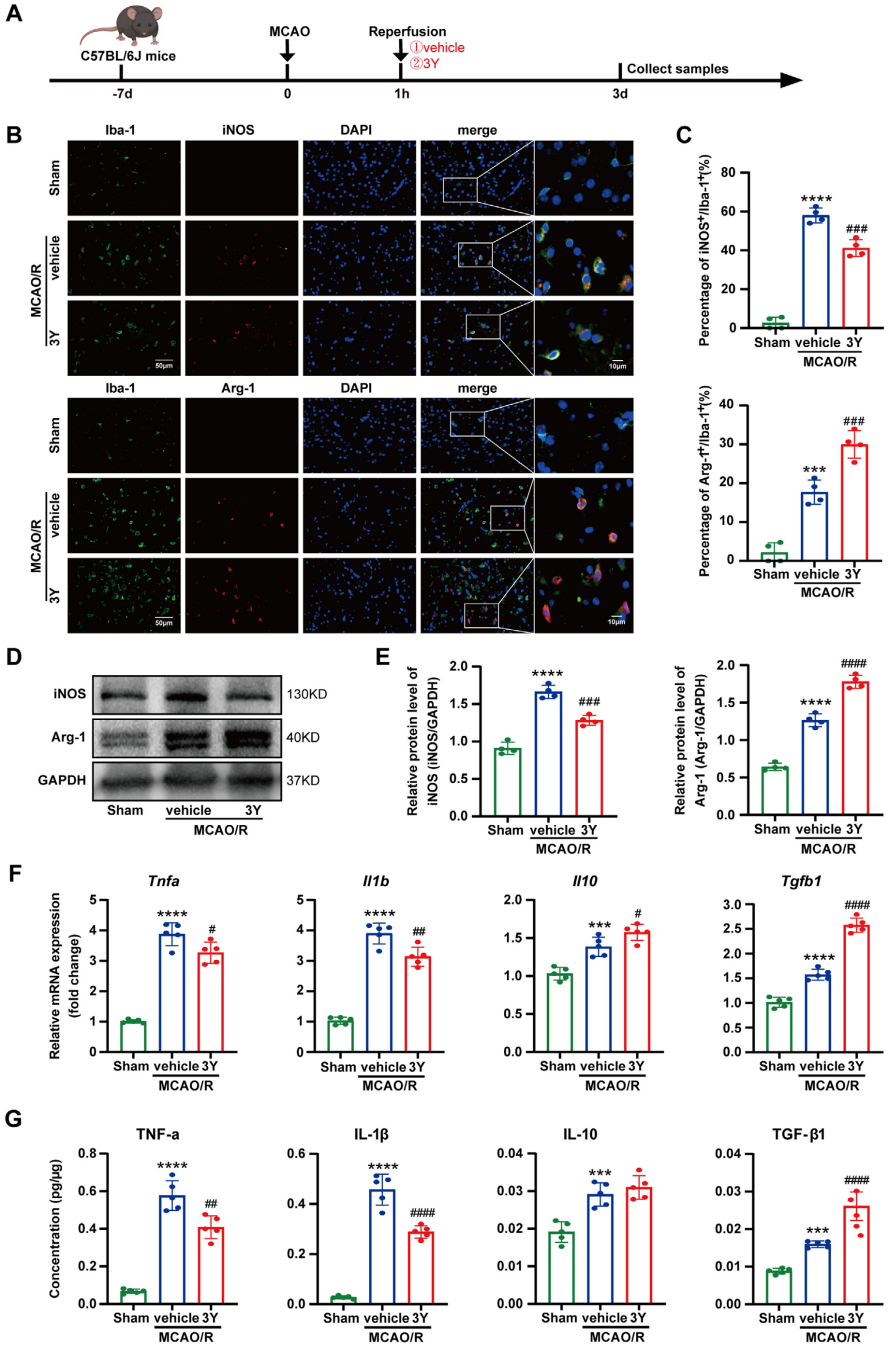
3Y promotes a protective microglial functional state and modulates associated cytokine expression following I/R injury. (A) The flow diagram of animal experiment. (B) Representative immunostaining photographs of microglial polarization by Iba‐1 (microglia marker, green), iNOS (a marker for pro‐inflammatory state, red), Arg‐1 (a marker for anti‐inflammatory state, red) and DAPI (nuclear marker, blue). Scale bar = 50 mm. (C) Quantitative analysis of the percentage of iNOS labeled cells to total Iba‐1 labeled cells and the percentage of Arg‐1 labeled cells to total Iba‐1 labeled cells. (*n* = 4/group). (D) Representative western blot of iNOS and Arg‐1. (E) The densitometric quantifications of iNOS and Arg‐1 (*n* = 4/group). (F) qRT‐PCR analyzes of mRNA levels of pro‐inflammatory cytokines (*Tnfa*, *Il1b*) and anti‐inflammatory cytokines (*Il10*, *Tgfb1*) in peri‐infarct region (*n* = 5/group). (G) ELISA analyzes of the expressions of matched cytokines (*n* = 5/group). Data were presented as the mean ± SEM and were analyzed by ANOVA. ***p < 0.001, ****p < 0.0001 versus Sham group; ^#^ *p* < 0.01, ^##^ *p* < 0.01, ^###^ *p* < 0.001, ^####^ *p* < 0.0001 versus MCAO/*R* + vehicle group.

Beyond its direct transcriptomic impact on neurons, our snRNA‐seq data offered a clue of a broader, indirect mechanism. We observed a significant increase in the proportion of microglia in the 3Y‐treated group (Figure 3G), prompting us to hypothesize that the 3Y‐salvaged neurons might actively shape the immune microenvironment. We therefore investigated whether 3Y influences post‐stroke neuroinflammation by modulating microglial functional states (Figure 4A). We assessed microglial functional phenotypes using specific markers: iNOS for the pro‐inflammatory state and Arg‐1 for the anti‐inflammatory state, along with associated cytokine profiling. As shown in Figure 4B,C, I/R injury increased the proportion of Iba‐1^+^ cells expressing iNOS (iNOS^+^/Iba‐1^+^) while decreasing the fraction of Arg‐1^+^/Iba‐1^+^ cells. Treatment with 3Y significantly reversed these changes, reducing iNOS^+^ cells and elevating Arg‐1^+^ cells among Iba‐1^+^ microglia compared to the vehicle group. These findings were corroborated by western blot analysis, which showed decreased iNOS and increased Arg‐1 protein levels following 3Y administration (Figure 4D,E). We further evaluated cytokine expression linked to microglial states. qRT‐PCR revealed that cerebral ischemia markedly elevated pro‐inflammatory cytokine mRNA levels, whereas 3Y treatment significantly reduced *Tnfa* and *Il1b* and enhanced expression of the anti‐inflammatory cytokines *Il10* and *Tgfb1* (Figure 4F). ELISA results confirmed that the vehicle group exhibited higher inflammatory cytokine release than the sham group, and 3Y administration significantly suppressed TNF‐α and IL‐1β secretion while boosting TGF‐β1 release. Although IL‐10 showed an upward trend, the difference was not significant (Figure 4G). Together, these results demonstrate that 3Y alleviates cerebral I/R injury by shifting microglia from a pro‐inflammatory toward an anti‐inflammatory functional state.

### 3.5 Peptide 3Y Modulated Microglia Polarization Toward Anti‐Inflammatory Phenotype Through cAMP Pathway

**FIGURE 5:**
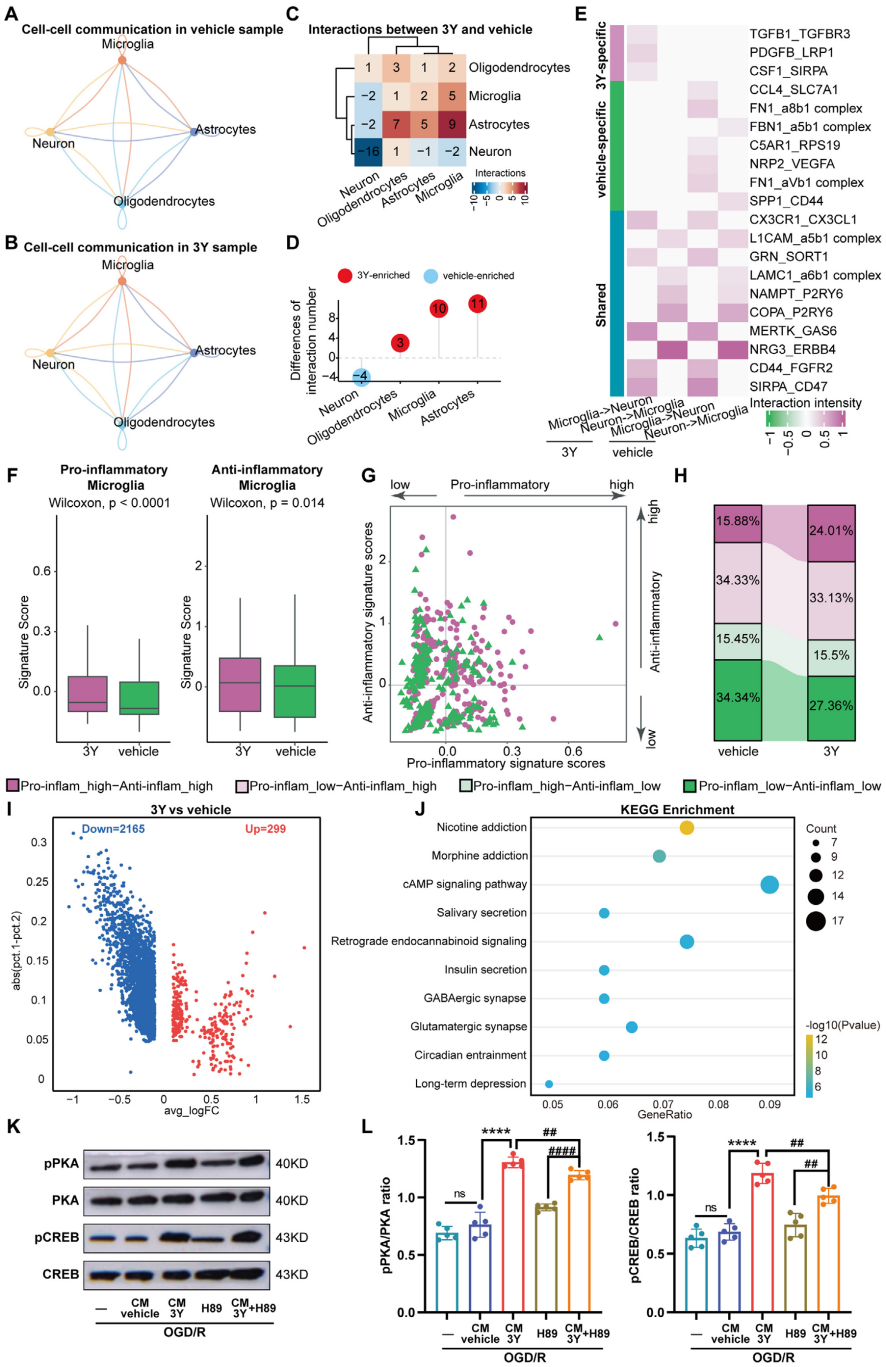
The influence of peptide 3Y on cell communication between neurons and microglia. (A) The cell–cell communication in vehicle‐treated sample. (B) The cell–cell communication in 3Y‐treated sample. (C) Matrix diagram of cell‐to‐cell interactions between 3Y and vehicle samples after ischemic stroke. (D) The difference of the number of ligand‐receptor interactions between microglia and other cell types in the vehicle‐ and 3Y‐treated groups. Red dot: 3Y‐treated enriched. Bule dot: Vehicle‐treated enriched. (E) Heatmap showing the interaction strength of ligand‐receptor pairs between neuron and microglia. (F) Comparison of microglial functional state scores for the damage‐associated (left) and protective (right) states between 3Y‐ and vehicle‐treated groups. (G) The distribution of microglia with four statuses. (H) Stacked histogram showing the percentage of four statuses of microglia. (I) Volcano plots of gene differential expression (log2FC > 0.15, FDR < 0.05) in microglia between 3Y and vehicle group. (J) Scatter plot of KEGG analysis of DEGs in microglia treated with peptide 3Y. (K) Western blots of the protein level in cAMP/PKA signaling pathway. (L) Quantification of p‐PKA/PKA, p‐CREB/CREB proteins in each group after OGD/R. Data were shown as the mean ± SEM (*n* = 5) and were analyzed by ANOVA. **p < 0.01, ***p < 0.001, ****p < 0.0001; ^##^ *p* < 0.01, ^###^ *p* < 0.001, ^####^ *p* < 0.0001.

Having demonstrated that 3Y promotes an anti‐inflammatory microglial phenotype in vivo, we leveraged our snRNA‐seq dataset to elucidate the potential mechanism of this reprogramming. To gain a comprehensive understanding of the intercellular communication between neurons and other cell types following cerebral I/R injury, we performed a high‐resolution dissection of cellular interactions using Cellphone DB on single‐cell RNA sequencing data from peri‐infarct tissues of 3Y‐ and vehicle‐treated mice after MCAO/R (Figure 5A,B). The analysis revealed a general reduction in ligand–receptor pairs between neurons and immune cells in the 3Y‐treated group compared to the vehicle group (Figure 5C), including notably diminished crosstalk between neurons and microglia (Figure 5D). Neuron–microglia crosstalk prevents microglial hyperactivation by minor stimuli [37], thereby contributing to the maintenance of their surveillant state [38]. Further investigation into neuron–microglia signaling identified several unique ligand–receptor interactions in the 3Y‐specific treated group, such as TGFB1‐TGFBR3, PDGFB‐LRP1, and CSF1‐SERPA increased remarkably, all of which have previously been reported to associate with macrophage phenotype polarization (Figure 5E). To explore this shift functionally, we scored microglia using gene sets representative of pro‐inflammatory and anti‐inflammatory states. While both scores were elevated in the 3Y group relative to vehicle (Figure 5F), subclustering microglia based on these scores (threshold zero; Figure 5G,H) revealed a clear redistribution: the proportion of microglia with an anti‐inflammatory signature increased from 50.21% in vehicle‐treated mice to 57.14% in the 3Y‐treated group (Figure 5H). Moreover, a distinct microglial subpopulation exhibiting high scores for both pro‐inflammatory and anti‐inflammatory genes expanded significantly following 3Y treatment (vehicle: 15.88%; 3Y: 24.01%), suggesting that 3Y may facilitate a functional transition within microglia toward an anti‐inflammatory state. Transcriptome analysis further identified 299 upregulated and 2165 downregulated genes in microglia from 3Y‐treated mice (Figure 5I). KEGG pathway enrichment highlighted significant associations with cAMP signaling and synaptic‐related processes—pathways previously implicated in regulating microglial activation states [39], and our present experiment also observed that microglia in the 3Y‐treated sample were enriched in the cAMP signaling pathway (Figure 5J). Next, we further carried out the functional validation in vitro. The results showed that 3Y obviously increased the protein level of phosphorylated PKA (pPKA) and phosphorylated CREB (pCREB) in microglia after OGD/R. However, the indirect facilitating effect of 3Y on the microglial cAMP signaling pathway was reversed by H89 (a widely used and highly selective PKA inhibitor) (Figure 5K), indicating that the anti‐inflammatory transformation of microglia mediated by 3Y is partially achieved via the cAMP‐PKA signaling pathway. Together, these results suggest that although 3Y reduces the overall quantity of neuron–microglia interactions, it qualitatively alters these communications to favor an anti‐inflammatory microglial phenotype, indicating that the neuroprotective effect of 3Y may be partly mediated through indirect modulation of microglial functional states.

**FIGURE 6:**
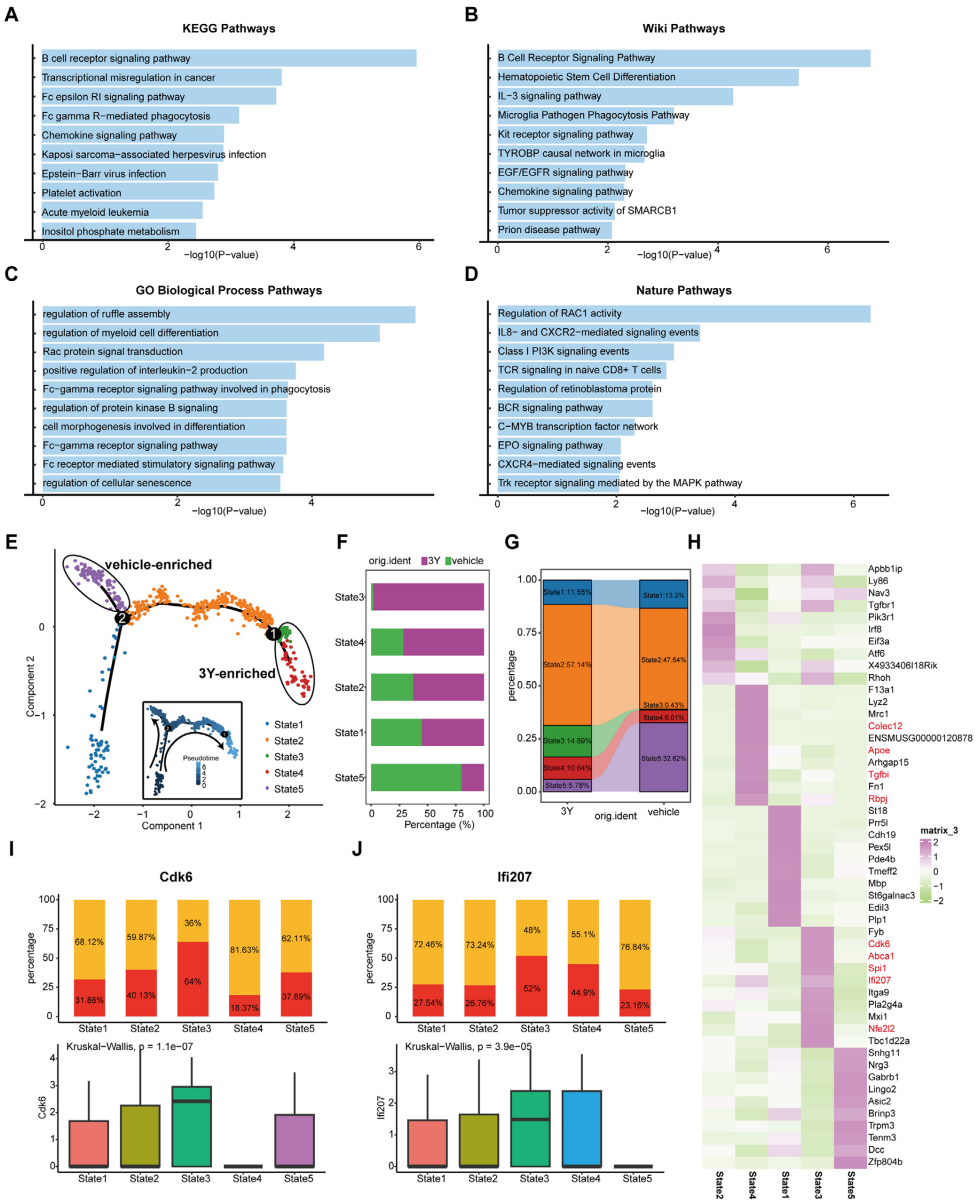
Unsupervised clustering analysis and functional enrichment analysis of all microglia from 3Y‐ and vehicle‐treated mice. (A–D) The top 10 pathways enriched in 3Y microglia‐upregulated genes from KEGG (A), Wiki (B), GO Biological Process (C) and Nature (D) databases. (E) Pseudotime trajectory of all microglia from 3Y‐ and vehicle‐treated mice. Cells were visualized with colors representing distinct States identified through unsupervised clustering in Monocle2. (F) Proportional histogram depicting the proportion of five states of microglia from 3Y‐ and vehicle‐treated mice. (G) Sankey diagram showing the proportion of the five states of microglia in 3Y‐ and vehicle‐ treated mice. (H) Heatmap showing the top 10 specific markers of each State. The red‐colored genes represent immune regulatory genes with anti‐inflammatory properties whether in macrophages, myeloid cells or glial cells. (I, J) The representative genes for significantly upregulated immunomodulatory anti‐inflammatory properties of 3Y‐ compared to vehicle‐ treated mice. Top: Bar plot showing the fraction of CDK6^+^ and CDK6^−^ cells across the five States. Bottom: Box plot showing the expression level of CDK6 across the five States. (J) Top: Bar plot showing the fraction of IFI207^+^ and IFI207^−^ cells across the five States. Bottom: Box plot showing the expression level of IFI207across the five States.

To further investigate the influence of 3Y on microglia, we performed functional enrichment analysis on the upregulated microglial genes following 3Y treatment. The results revealed that these upregulated genes were primarily enriched in biological processes such as cyclin regulation, immunoregulation, myeloid reprogramming, and macrophage‐mediated modulation of the tumor microenvironment (Figure 6A–D). Subsequently, we conducted unsupervised clustering and pseudotime analysis on all microglial cells, which classified the cells into five distinct states based on gene expression patterns (Figure 6E). The 3Y‐treated samples were predominantly clustered in State 3 and State 4, whereas the vehicle‐treated samples were mainly located in State 5, with States 1 and 2 regarding as the shared populations (Figure 6F,G). Deeper analysis of the genes enriched in States 3 and 4 indicated that these genes were primarily involved in immunoregulation [40], cell migration and differentiation [41], tissue repair [42] and the inflammatory regulation of macrophages mediating tumor immune escape [43] (Figure 6H). We also discovered that CDK6 and IFI207 are the immune regulatory genes with anti‐inflammatory properties that are significantly upregulated in 3Y‐enriched states (Figure 6I,J). These results suggested that 3Y‐treated microglia tend to shift toward protective phenotypes, such as immunoregulatory, neuroprotective or anti‐inflammatory. Taken together, these findings support the conclusion that 3Y treatment may exert stroke‐resistant effects by regulating the phenotypic transformation of microglia indirectly.

### 3.6 3Y‐Treated Neuronal Conditioned Medium Reprogramed Microglia Toward a Protective State In Vitro

**FIGURE 7:**
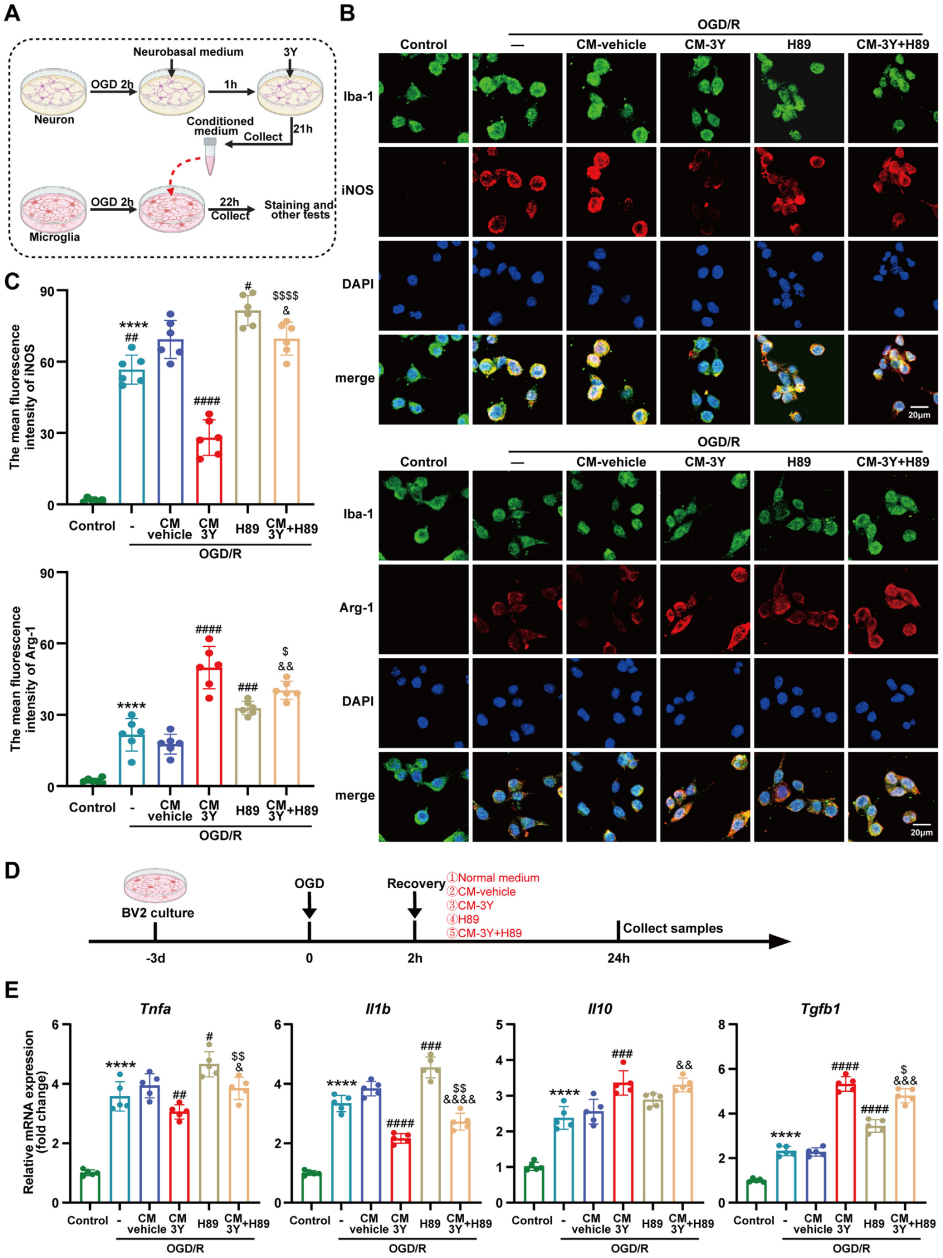
Effects of neuron‐derived conditioned medium on the functional state of BV2 microglia after OGD/R. (A) Diagram of neuronal conditioned culture medium extraction and microglia intervention. (B) Representative pictures of double immunofluorescence staining of BV2 microglial polarization. Scale bar = 20 mm. (C) The quantitative analysis of fluorescence intensity of iNOS and Arg‐1 using ImageJ software (*n* = 6). (D) Schematic diagram of the cell experimental design. (E) The mRNA expression levels of inflammatory cytokines (*n* = 5). ****p < 0.0001 versus Control group. ^#^ *p* < 0.05, ^##^ *p* < 0.01, ^###^ *p* < 0.001, ^####^ *p* < 0.0001, versus OGD/*R* + CM‐vehicle group. ^$^ *p* < 0.05, ^$$^ *p* < 0.01, ^$$$$^ *p* < 0.0001 versus OGD/*R* + CM‐3Y group. ^&^ *p* < 0.05, ^&&^ *p* < 0.01, ^&&&^ *p* < 0.001, ^&&&&^ *p* < 0.0001, versus OGD/*R* + H89 group. Data are presented as the mean ± SEM from at least three independent experiments.

Our snRNA‐seq analysis predicted that 3Y qualitatively reshapes neuron‐to‐microglia signaling. To directly test the causality of this prediction—that the salvaged neurons themselves instruct microglial polarization—we performed an in vitro co‐culture system using conditioned medium (CM). In this study, iNOS and Arg‐1 were utilized as functional markers to identify microglia in pro‐inflammatory and anti‐inflammatory states, respectively. To investigate whether the secretome of 3Y‐treated hypoxic neurons modulates the phenotypic switching of microglia from a pro‐inflammatory to an anti‐inflammatory state, we exposed OGD‐injured BV2 cells at reoxygenation to conditioned medium (CM) derived from OGD/R‐stimulated HT22 cells under different treatments (Figure 7A). Immunofluorescence analysis indicated that OGD/R provoked a mixed response in BV2 microglia, evidenced by the simultaneous induction of the pro‐inflammatory marker iNOS and the anti‐inflammatory marker Arg‐1. However, the functional state of these microglia was significantly shifted toward an anti‐inflammatory phenotype when exposed to CM from 3Y‐treated HT22 cells, as evidenced by a pronounced decrease in iNOS and an increase in Arg‐1 intensity compared to the vehicle‐CM group. On the contrary, the treatment with H89 showed the opposite results, with higher intensity of iNOS and lower intensity of Arg‐1 when compared with the vehicle‐CM group; nevertheless, the addition of 3Y‐CM partially reversed the adverse effects of H89 (Figure 7B,C). The inflammatory profile of microglia can serve as a key readout for their functional state (Figure 7D). Compared to the CM‐vehicle group, CM‐3Y significantly diminished the increased mRNA level of pro‐inflammatory cytokines (*Il1b* and *Tnfa*) and further enhanced the mRNA level of the anti‐inflammatory cytokine (*Il10* and *Tgfb1*) in OGD/R‐injured BV2 microglia, while the inhibitory effect of PKA inhibitor H89 on the mRNA levels of inflammatory cytokines can be effectively reversed by CM‐3Y (Figure 7E). Notably, compared to BV2 cells maintained in normal medium, exposure to CM‐vehicle significantly upregulated the expression of the pro‐inflammatory marker iNOS (Figure 7C) and the proinflammatory cytokine *Il1b* at the mRNA level (Figure 7E). In contrast, CM‐vehicle had no significant effect on markers associated with an anti‐inflammatory state or on anti‐inflammatory cytokines. These results indicate that the neuronal microenvironment shaped by peptide 3Y can skew microglial functional states, attenuating the pro‐inflammatory‐related response in BV2 microglia following OGD/R, and the protective effect of 3Y is closely related to the activation of the cAMP signaling pathway.

## 4 Discussion

Building on our foundational discovery that Syt3 exacerbates ischemic injury via AMPAR regulation [11], this work elucidates a groundbreaking dual‐mechanism of its inhibitor, peptide 3Y. The neuroprotection extends beyond the direct suppression of neuronal apoptosis to include an indirect, yet critical, pathway: the phenotypic switching of microglia from a pro‐inflammatory to an anti‐inflammatory state, as revealed by snRNA‐seq analysis of the penumbra.

To elucidate the molecular mechanisms underlying the functional recovery, we first employed the widely accepted MCAO/R model to investigate the therapeutic potential of peptide 3Y. Treatment with 3Y resulted in a marked improvement in neurological deficits, confirming that inhibition of Syt3 internalization promotes significant functional recovery. Subsequent snRNA‐seq data demonstrated that the neuroprotective effect of 3Y is fundamentally mediated through the suppression of neuronal apoptosis. This conclusion is supported by a global transcriptomic shift toward an anti‐apoptotic state in neurons, characterized by the downregulation of key pro‐apoptotic mediators (Bax, Casp3, Casp9), upregulation of the anti‐apoptotic gene Bcl‐2 [44], and enrichment of pro‐survival signaling pathways such as MAPK [45]. These findings align with extensive prior research on post‐stroke neuronal death, which establishes apoptosis as the predominant mode of cell death in the ischemic penumbra, with the Bax/Bcl‐2 ratio and Caspase‐3 activation serving as its hallmarks [46]. Furthermore, pathways like MAPK and Akt are well‐documented as critical for neuronal survival under ischemic stress [47, 48]. The concordance of our results with these established mechanisms provides a solid theoretical foundation for the efficacy of 3Y.

Although targeting neuronal apoptosis has been a long‐standing direction in stroke therapy [49], clinical translation has repeatedly encountered setbacks, partly because many strategies (e.g., broad‐spectrum caspase inhibitors) act downstream in the apoptotic pathway, interfering with normal physiological processes [50]. The innovativeness of this study lies in the first revelation that inhibiting Syt3 internalization represents a unique upstream mechanism for achieving an anti‐apoptotic effect. This approach distinguishes itself from other upstream strategies targeting excitotoxicity, such as NMDA receptor antagonists which often lead to debilitating side effects in clinical trials due to their broad disruption of synaptic transmission [51]. In contrast, by specifically uncoupling Syt3‐mediated AMPAR endocytosis from calcium overload, 3Y appears to fine‐tune the pathological excitatory signaling without completely abolishing the physiological synaptic function, offering a potentially wider therapeutic window. Syt3 directly responds to calcium overload by regulating AMPAR internalization [11], and our results indicate that inhibiting this process effectively mobilizes endogenous survival pathways. Unlike directly using apoptosis inhibitors, 3Y intervenes in the AMPAR internalization process downstream of excitotoxicity, blocking the initiation of apoptotic signals at a more proximal point. Previous studies have confirmed that the synthetic peptide Tat‐GluA2‐3Y is a specific inhibitor of the clathrin‐mediated endocytosis of GluA2‐containing AMPARs [52], and the 3Y motif of GluA2 is the competitive binding site for Syt3 binding to AMPARs [6], which indirectly supports the potential of 3Y as a neuroprotective agent. This conclusion was validated in animal models: 3Y treatment reduced levels of pro‐apoptotic proteins, the proportion of TUNEL‐positive neurons, and significantly decreased the number of degenerating cells labeled with FJC [53]. All evidence indicates that restricting Syt3 endocytosis by peptide 3Y reduces calcium overload and neuron excitotoxicity, and consequently exerts neuroprotective effects following I/R injury by alleviating neuron degeneration and apoptosis via inhibiting apoptotic signals.

However, the protective story of 3Y appears to be more complex than solely bolstering neuronal intrinsic resilience. Thus, while our findings establish that 3Y directly bolsters neuronal resistance to apoptosis by inhibiting Syt3 internalization, our work reveals a more profound insight: these salvaged neurons play an active role in remodeling their surrounding microenvironment. Our findings uncover a non‐canonical pathway of immunomodulation where peptide 3Y ameliorates neuroinflammation by fostering a protective neuronal niche. 3Y modulates microglia within penumbra by decreasing the expression of the classical pro‐inflammatory marker iNOS as well as pro‐inflammatory cytokines TNF‐α and IL‐1β, while increasing the levels of the neuroprotective marker Arg‐1 and the anti‐inflammatory factor TGF‐β1 in MCAO/R mice. However, the discordance between the elevated IL‐10 mRNA and the non‐significant increase in protein from our data mainly attributes to the local cerebral environment alterations following ischemic–hypoxic injury, which may lead to aberrant transcription and translation of IL‐10. According to snRNA‐seq analysis, the reduced proportion of microglia in the vehicle‐treated group is attributable to enlarged infarct volume causing direct microglial death [54], whereas 3Y administration significantly increased the proportion of microglia surrounding the ischemic core, likely as a consequence of the overall tissue preservation and a more viable microenvironment resulting from its anti‐apoptotic effect. Rather than reducing all communication, 3Y refines it, enhancing signaling quality to promote an anti‐inflammatory microglial phenotype despite a quantitative reduction in interactions. This alteration in cellular crosstalk is context‐dependent, as research shows that the strength of interactions is determined by specific infarct conditions or reperfusion time after stroke [55]. In our present data, three ligand‐receptor pairs were significantly upregulated in 3Y‐treated samples, and all these ligand‐receptor pairs were related to the regulation of immune inflammation as reported in previous studies. In particular, TGFB1‐TGFBR3 is involved in the polarization of macrophages [56], while CSF1 directly participates in regulating the inflammatory response of microglia [57]. Interestingly, our finding that 3Y reduces neuron–microglia ligand‐receptor pairs appears inconsistent with a prior study using a novel nanomedicine delivery of Fingolimod hydrochloride, which reported an increase [58]. This discrepancy may underscore 3Y's unique mechanism: it does not merely enhance all communication but qualitatively “reprograms” it. Although overall interaction was weakened, this change was conducive to the reprogramming of microglia toward specific subsets. Furthermore, KEGG analysis of DEGs in 3Y‐treated microglia showed significant enrichment in the cAMP signaling pathway. Certainly, our in vitro experiments also confirmed that the positive regulation of 3Y on cAMP pathway was limited when the cAMP signal was inhibited by the specific inhibitor of PKA. This indicates that the influence of 3Y on microglia is achieved through the cAMP signaling pathway, which is closely associated with microglial phenotypic switching [39]. This offers compelling support for the pivotal role of neuro‐immune dialogue in repair. While pathways like CD200‐CD200R illustrate how viable neurons maintain microglial quiescence [59], we demonstrate that 3Y‐fortified neurons actively assume the role of a pivotal regulator of local immunity, for which the results of our further unsupervised clustering and pseudotime analysis of microglia provided strong evidence. 3Y‐treated microglia are more inclined to transform toward an immunoregulatory and tissue repair‐oriented direction for improving the local microenvironment [40, 41, 42], resonating with the principle of “protective autoimmunity” [60].

Crucial causal evidence came from in vitro experiments: conditioned medium from 3Y‐treated, hypoxic neurons was sufficient to skew microglia toward an anti‐inflammatory and neuroprotective direction, although this phenomenon was partially weakened by PKA‐specific inhibitor. And significantly, the anti‐inflammatory and neuroprotective effects of peptide 3Y have been corroborated in vivo by immunohistochemistry and cytokine profiling. This indicates that salvaged neurons actively instruct microglia to adopt a protective functional state, constituting a sophisticated two‐pronged therapeutic mechanism. The altered secretome of 3Y‐treated neurons is capable of reprogramming microglia. This demonstrates that 3Y's neuroprotection is a bidirectional process: it directly enhances neuronal survival resilience and indirectly creates a supportive microenvironment by improving neuro‐immune communication. This “two‐birds‐with‐one‐stone” effect likely underlies its significant efficacy. The apparent paradox of a “quantitative reduction but qualitative enhancement” in interactions may reflect more precise cellular crosstalk. Under severe injury, “distress signals” released by numerous stressed neurons may exacerbate inflammation [61]. By reducing the population of dying neurons, 3Y potentially diminishes this environmental “noise,” allowing the homeostatic “instructional” signals from healthier neurons to prevail—a key feature of 3Y's microenvironmental regulation. Thus, 3Y's strategy is distinct: it bypasses direct immunosuppression by augmenting intrinsic neuronal health, effectively enabling neurons to dictate a beneficial immune outcome—reconstructing the surrounding microenvironment, promoting microglia toward an anti‐inflammatory or tissue repair oriented direction by activating the cAMP‐PKA signaling pathway, and ultimately alleviating the inflammatory response—a potentially more precise and sustainable therapeutic approach. Collectively, our findings establish a novel neuro‐immune axis wherein 3Y, by rescuing neurons, empowers them to orchestrate a protective immune response.

Certainly, our study has limitations that also chart a course for future investigation. Firstly, the specific neuroprotective factors within the 3Y‐modified neuronal secretome that are necessary and sufficient for microglial reprogramming remain to be pinpointed. Their identification via proteomic profiling constitutes a critical next step. Secondly, while our work has primarily focused on the neuron–microglia axis, the role of Syt3 in other cerebral cell types following stroke, as well as whether 3Y possesses a broader therapeutic time window, constitutes important avenues for future exploration. Thirdly, primary cells play an irreplaceable and significant role in the study of brain functions. Future research should adopt. Finally, considering the neuron‐specific localization of Syt3, it cannot be completely ruled out that the low expression possibility of Syt3 in other types of cells or be induced under specific pathological conditions. In the future, the use of cell type‐specific knockout techniques or high‐resolution single‐cell sequencing will help to thoroughly clarify the cellular expression profile of Syt3, which is of great value for in‐depth exploration of clinical translation challenges, such as treatment time windows, optimal doses, and blood–brain barrier penetration.

## 5 Conclusions

In conclusion, our study demonstrates that the peptide 3Y, by inhibiting Syt3 internalization, confers robust neuroprotection against cerebral I/R injury. This protection is mediated through a dual mechanism: a direct anti‐apoptotic effect on neurons and an indirect immunomodulatory effect on microglia. We provide multi‐faceted evidence from snRNA‐seq, in vivo models, and in vitro co‐culture systems showing that the neurons rescued by 3Y release factors that actively reprogram microglia, promoting it to reach a protective state with anti‐inflammatory or immune‐modulating functions. Our work thus identifies Syt3 as a promising novel target for stroke therapy and elucidates a key mechanism by which neuroprotection and immunomodulation are functionally linked in the injured brain.

## Author Contributions

All authors guaranteed the integrity of the entire research. Q.X. and J.C. designed the experiment. H.X., H.M., R.C., and T.G. conducted the experiments. Data were analyzed by F.H. and Y.Y. Manuscript was drafted by H.X. and Q.X. All authors had read and approved the manuscript.

## Funding

This work was supported by the Natural Scientific Foundation of China (Grant 82371365), Changzhou Health and Youth Talent Training Project (Grant CZQM2023029), Changzhou Sci & Tech Program (Grants CJ20253002 and CJ20220001) and Medical Education Collaborative Innovation Fund of Jiangsu University (Grant JDYY2023044).

## Ethics Statement

The study was approved and conducted in compliance with the Ethics Committee of Soochow University (approval number: SUDA20240412A01).

## Conflicts of Interest

The authors declare no conflicts of interest.

## Supporting information


**Table S1:** Primer sequences for qRT PCR.

**Table S2:** The total number of cells collected from vehicle‐ and 3Y‐treated.

## Data Availability

All the data supporting the findings of this study are available on request from the corresponding author.
