# Supplementary material for: Restricting Synaptotagmin‐3 Internalization Mitigates Cerebral Ischemia/Reperfusion Injury by Curtailed Neuronal Apoptosis and Microglial Re‐Programming
Source: CNS Neurosci Ther. 2026 Mar 5;32(3):e70815. doi: 10.1002/cns.70815 (PMC12963021; doi:10.1002/cns.70815)
Supplement: Supplementary file 1 — Table S1: Primer sequences for qRT PCR. [file CNS-32-e70815-s001.docx]

Supplementary Table 1

Primer sequences for qRT PCR

| Gene | Forward (5´-3´) | Reverse (5´-3´) |
| --- | --- | --- |
| *Tnfa* | GTCTACTGAACTTCGGGGTGA | ATGATCTGAGTGTGAGGGTCTG |
| *Il1b* | GCAACTGTTCCTGAACTCAACT | ATCTTTTGGGGTCCGTCAACT |
| *Il10* | CAGAGCCACATGCTCCTAGA | TGTCCAGCTGGTCCTTTGTT |
| *Tgfb1* | ACTGGAGTTGTACGGCAGTG | GGGGCTGATCCCGTTGATTT |
| *Gapdh* | CATGGCCTTCCGTGTTCCTA | GCCTGCTTCACCACCTTCTT |
